# Complex Gene Regulation Underlying Mineral Nutrient Homeostasis in Soybean Root Response to Acidity Stress

**DOI:** 10.3390/genes10050402

**Published:** 2019-05-27

**Authors:** Qianqian Chen, Weiwei Wu, Tong Zhao, Wenqi Tan, Jiang Tian, Cuiyue Liang

**Affiliations:** Root Biology Center, State Key Laboratory for Conservation and Utilization of Subtropical Agro-bioresources, College of Natural Resources and Environment, South China Agricultural University, Guangzhou 510642, China; qian2016200202@163.com (Q.C.); weiweiwu2016@163.com (W.W.); zhaotong7989@gmail.com (T.Z.); xiao_tantx@163.com (W.T.); jtian@scau.edu.cn (J.T.)

**Keywords:** soybean, roots, mineral nutrient, transporter, acidity stress

## Abstract

Proton toxicity is one of the major environmental stresses limiting crop production and becomes increasingly serious because of anthropogenic activities. To understand acid tolerance mechanisms, the plant growth, mineral nutrients accumulation, and global transcriptome changes in soybean (*Glycine max*) in response to long-term acidity stress were investigated. Results showed that acidity stress significantly inhibited soybean root growth but exhibited slight effects on the shoot growth. Moreover, concentrations of essential mineral nutrients were significantly affected by acidity stress, mainly differing among soybean organs and mineral nutrient types. Concentrations of phosphorus (P) and molybdenum (Mo) in both leaves and roots, nitrogen (N), and potassium (K) in roots and magnesium (Mg) in leaves were significantly decreased by acidity stress, respectively. Whereas, concentrations of calcium (Ca), sulfate (S), and iron (Fe) were increased in both leaves and roots. Transcriptome analyses in soybean roots resulted in identification of 419 up-regulated and 555 down-regulated genes under acid conditions. A total of 38 differentially expressed genes (DEGs) were involved in mineral nutrients transportation. Among them, all the detected five GmPTs, four GmZIPs, two GmAMTs, and GmKUPs, together with GmIRT1, GmNramp5, GmVIT2.1, GmSKOR, GmTPK5, and GmHKT1, were significantly down-regulated by acidity stress. Moreover, the transcription of genes encoding transcription factors (e.g., GmSTOP2s) and associated with pH stat metabolic pathways was significantly up-regulated by acidity stress. Taken together, it strongly suggests that maintaining pH stat and mineral nutrient homeostasis are adaptive strategies of soybean responses to acidity stress, which might be regulated by a complex signaling network.

## 1. Introduction

Acid soils with pH lower than 5.5 comprise up to approximate 30% of total areas of the planet and one-half of world’s potentially arable lands [1,2,3,4]. In the tropics and subtropics, soil acidification is a natural and very slow process. However, anthropogenic activities, such as inappropriate application of fertilizers and massive use of fossil energy sources, have accelerated soil acidification in recent time [5,6,7,8,9].

In acid soils, H^+^ rhizotoxicity is recognized as a major limiting factor for crop production [2]. Field experiments in Northern Idaho showed that yield of important crops decreased on even moderately low-pH soils without Al toxicity [10]. It has been suggested that physiological response of plant roots to low-pH stress could occur in a very short time period. For example, root cells of alfalfa (*Medicago sativa*) and Arabidopsis (*Arabidopsis thaliana*) lost viability within four and two-hour exposure to low pH, respectively [11,12]. In *Lotus corniculatus*, the root growth was obviously inhibited after 5 d of low-pH treatment [13]. Further results showed that low-pH stress inhibited plant root growth through complex mechanisms such as affecting root water conductance, changing plasma membrane Ca^2+^ fluctuation, disturbing root gravitropic responses, disrupting cell wall polysaccharide stability and changing cytosol pH homeostasis [12,13,14,15,16,17,18,19,20]. 

Accompanied with root growth inhibition, low-pH stress can disturb plant mineral nutrient acquisition mainly through destruction of proton gradients across plasma membranes [14,15,16,21,22,23,24,25]. One typical example is the nitrate (NO_3_^−^) up-take in plant roots. NO_3_^−^ is taken-up by either nitrate/peptide transporter family (NPF/NRT1) or the nitrate/proton symporter family (NRT2) [26,27]. Therefore, the transport of NO_3_^−^ across the plasma membrane in root epidermal cells significantly influences the rhizosphere pH, and vice versa, the rhizosphere pH can alter plant NO_3_^−^ uptake [22,23,24,28,29,30]. For instance, evidences showed that the proton stress enhanced the NO_3_^−^ uptake mediated by NRT1.1 in Arabidopsis and caused significant rhizosphere alklification [24]. Moreover, another NO_3_^−^ transporter OsNRT2.3b locates on the plasma membrane and has a regulatory motif on the cytosolic side that acts to switch NO_3_^-^ transport activity on or off by a pH-sensing mechanism [31]. These results suggest that external pH could affect the plant NO_3_^−^ sensing and in turn improve plant adaptation to rhizosphere pH by the regulation of NO_3_^−^ transport [32]. 

With the aid of genome wide transcriptomic analysis and functional characterization of sets of genes, molecular mechanisms underlying complex responses of plants to acidity stress have been elucidated [33,34,35]. A microarray assay on wheat (*Triticum aestivum*) identified 1057 differentially expressed genes (DEGs) after two hours of acid treatment, in which rhizosphere alkalification was suggested to be the earliest responses to acidity stress [34]. Moreover, longer acid treatment (8 h) on Arabidopsis identified a total of 881 genes exhibited at least 2-fold changes in transcripts, suggesting redundancies and interactions among the responses to pH, auxin and pathogen elicitors [33]. Interestingly, a recent RNA-seq analysis in soybean found a total of 3242 genes were up-regulated by pH3.0 medium for 9 days. Among them, 27 genes were identified to be involved in the biosynthesis of glycollins, which are isoflavonoid-derived pathogen-inducible defense metabolites [35]. Moreover, genes function in systemic acquired resistance (SAR), which is a component of the plant immune system to pathogen, were also significantly up-regulated [35]. These results indicated that acidity stress is an elicitor of plant defensive responses to pathogens. Except for transcriptomic assays, complex regulatory mechanisms underlying plant acid tolerance have been widely documented through identification and functional analysis of STOP1 (*Sensitive to Proton Rhizotoxicity* 1) and its down-stream genes such as GAD (*Glutamate Decarboxylase*), ME (*Malic Enzyme*) and GDH (*Glutamate Dehydrogenase*) [36,37,38,39,40,41,42,43]. However, most of these studies mainly focus on the mechanisms underlying plant root growth adaptation to acidity stress, few studies have tried to investigate the effects of acidity stress on mineral nutrient acquisition and translocation in plants, especially in crops.

Soybean (*Glycine max*) is one of the most economically important leguminous crops, which provides more than a quarter of the world’s protein for food and animal feed [44]. Approximate 2000 years ago, soybean cultivation was expanded worldwide, and domesticated with wide adaptation to different soil types, especially acid soils [45,46]. For instance, in Brazil, the world’s second largest soybean producer, soybean is mainly grown on savannahs regions, which comprises prevalence of acid infertile soils with low soil pH, high aluminum (Al) availability, and low mineral nutrient-holding capacity [47,48]. Although intensive studies have been conducted to investigate soybean adaptation to Al toxicity and mineral nutrient deficiencies (e.g., phosphorus and nitrogen) on acid soils [42,49,50,51,52,53,54], few studies have been carried out to elucidate complex mechanisms underlying soybean adaptation to proton toxicity through integration of mineral nutrients accumulation and transcriptomic analyses. Therefore, the present study investigated the effects of acidity stress on soybean growth and mineral nutrients accumulation. Subsequently, transcriptome analysis was conducted to identify DEGs in soybean roots responding to acidity stress, which highlighted transcriptional regulatory mechanisms underlying its adaptive strategies, especially for mineral nutrient acquisition and translocation.

## 2. Materials and Methods

### 2.1. Plant Material and Growth Conditions

Soybean genotype Huachun 6 was used in the study. Soybean seeds were surface-sterilized with 10% (*v*/*v*) H_2_O_2_, then directly sown in the prepared peat soils (JIFFY, Hoek van Holland, The Netherlands) moistened with nutrient solution, of which soil pH was adjusted to 4.2 and 5.8 by addition of diluted H_2_SO_4_. After seed germination, seedlings were watered with deionized water every day and supplied with 200 mL nutrient solution with pH of 4.2 and 5.8 once a week. The nutrient solution was composed of (in mM): 1.5 KNO_3_, 1.2 Ca(NO_3_)_2_, 0.4 NH_4_NO_3_, 0.025 MgCl_2_, 0.5 MgSO_4_, 0.3 K_2_SO_4_, 0.3 (HN_4_)_2_SO_4_, 0.0015 MnSO_4_, 0.0015 ZnSO_4_, 0.0005 CuSO_4_, 0.00015 (NH_4_)_6_Mo_7_O_24_, 0.0025 H_3_BO_3_, 0.25 KH_2_PO_4_ and 0.04 Fe-Na-EDTA. After 30 days, plant dry weight, leaf number, shoot height, branch number, along with total root length, average root diameter, and root surface area were determined. Plants were also harvested for the determination of mineral nutrient concentrations of leaves and roots. All experiments had five biological replicates, each of which had one plant. 

For transcriptome analysis, soybean seeds were surface-sterilized and germinated in paper rolls moistened with half-strength nutrient solution as mentioned above. After germination, uniform seedlings were transplanted to hydroponic culture, the component of which was the same as mentioned above with pH 5.8 and pH 4.2, respectively. The solution pH was adjusted every day with diluted KOH or H_2_SO_4_ and renewed once a week. After 30 days, soybean roots were harvested for transcripteomic assay. Each replicate had two plants and was used for one cDNA library construction. Similarly, roots of other two plants in each replicate were harvested for quantitative real-time PCR (qRT-PCR) analyses. Meanwhile, dry weight, leaf number, shoot height, branch number, along with total root length, average root diameter, root surface area and mineral nutrient concentrations in roots of another two plants in each replicate were determined. All experiments had three replicates.

### 2.2. Determination of Mineral Nutrient Concentration 

Measurement of N and P concentrations in soybean samples was conducted using the continuous flowing analyzer (Series SA1100, SKALAR, Breda, The Netherlands). Briefly, about 0.1 g dry samples were incubated with 1 mL H_2_O_2_ and 5 mL H_2_SO_4_ overnight. The processed samples were then heating digested and constant-volumed to 50 mL. The resulted products were analyzed following the instruction in the manuals. 

The other dry samples were digested using the START D microwave digestion system (Series: 131825, Milestone, Sorisole, Italy) following the instruction in the manuals. Briefly, samples were incubated with 8 mL diluted HNO_3_ and 2 mL 30% H_2_O_2_ for overnight. The resulting products were applied to microwave digestion following the consecutive procedures, in which the temperature was raised to 130 ℃ within 10 min, then hold for 5 min at 130 ℃. After that, the temperature was raised to 180 ℃ within 10 min, and then hold for 30 min at 180 ℃ and ventilation for 30 min. After microwave digestion, mixtures were moved to plastic volumetric flasks and adjusted to 100 mL, then filtered with filter papers. The products were subsequently used for determination of potassium (K), calcium (Ca), magnesium (Mg), iron (Fe), molybdenum (Mo), zinc (Zn), copper (Cu), and manganese (Mn) by atomic absorption spectrometer (Series: ZA-3300, Hitachi, Tokyo, Japan), and sulfate (S) by inductively couple plasma atomic emission spectrometer (Series: 710-ES, Agilent, Santa Clara, CA, USA).

### 2.3. Transcriptome Analysis of Soybean Roots

Total RNA was extracted from soybean roots under acid (pH 4.2) and normal (pH 5.8) conditions using an RNeasy Mini Kit (Qiagen, Hilden, Germany). Then, the total RNA was digested with RNase free DNAase I (Qiagen, Hilden, Germany) for the purification of mRNA using Oligo (dT) magnetic beads. Purified mRNA was broken into short fragments and used for the cDNA synthesis. Short fragments were purified with a QiaQuick PCR Extraction Kit (Qiagen, Hilden, Germany) and connected with sequencing adapters. The proper products were selected for PCR amplification and cDNA library construction. The library was sequenced using BGISEQ500 (The Beijing Genomics Institute, Beijing, China). After sequencing, the low-quality, adaptor-polluted and high content of unknown base reads were filtered from the raw data. The clean reads were mapped to soybean reference genome (Glycine max Williams82.a2.v1) to predict novel transcripts. Subsequently, the coding novel transcripts were merged to get a complete reference, which was then used for the analysis of differentially expressed genes (DEGs). During the processes, software including HISAT, StringTie, Bowtie2 and RSEM was used to perform genome mapping, reconstruct transcripts, map clean reads to reference and calculate gene expression level, respectively [55,56,57]. Finally, DEseq2 was used to identify DEGs by different treatments with *p* value less than 0.05, and log_2_ Fold change 1 or-1 as the threshold. The transcriptomic data was up-loaded to GEO repository (https://www.ncbi.nlm.nih.gov/geo/query/acc.cgi?acc = GSE129320) with number of GSE129320. 

### 2.4. Quantitative Real-Time PCR Analysis

Total RNA was extracted from soybean roots using the RNA-solve reagent (OMEGA Bio-tek, Norcross, GA, USA). After being treated with RNase-free DNase I (Invitrogen, Carlsbad, CA, USA), total RNA was used to synthesize the first-strand cDNA using MMLV-reverse transcriptase (Promega, Madison, WI, USA) following instructions in manuals. The qRT-PCR analysis was conducted using SYBR Green-monitored on a ABI StepOne Plus real-time PCR system (Thermo Fisher Scientific, Waltham, MA, USA). Primer pairs (Table S1) for qRT-PCR were designed. Housekeeping gene *GmEF-1a* (accession no. X56856) was used as an internal control to normalize the expression of corresponding genes in soybean.

### 2.5. Statistical Analysis

All data were analyzed by Student’s t-test using SPSS software (IBM SPSS Statistics, Chicago, IL, USA).

## 3. Results

### 3.1. Effects of Low-pH Stress on Soybean Growth in Soils

**Figure 1:**
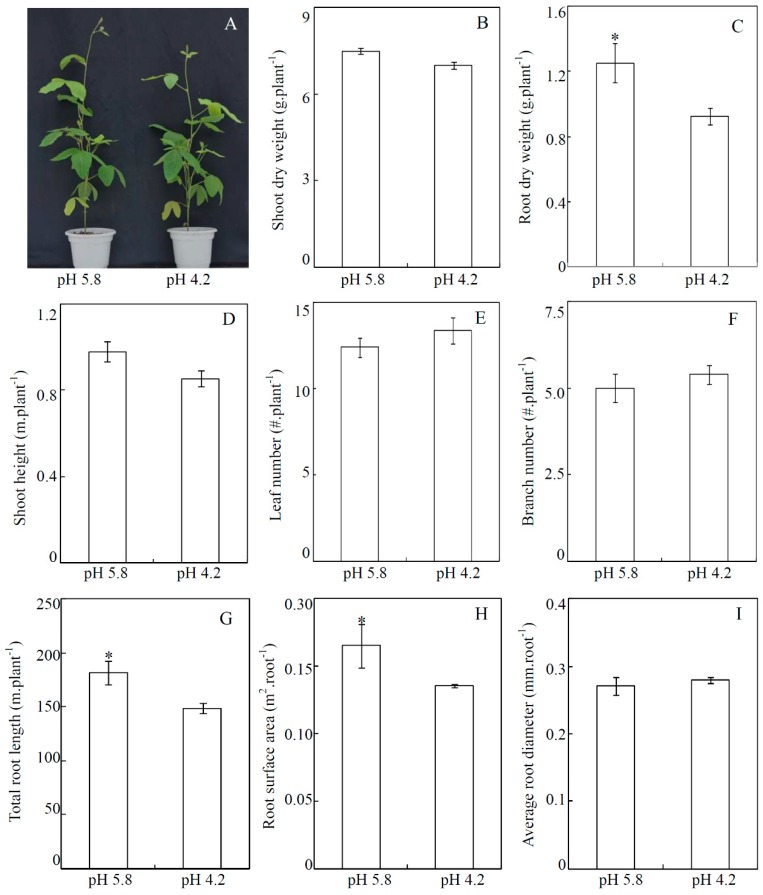
Effects of low-pH stress on soybean growth in soil culture. (**A**) Phenotype of soybean plants at two pH-treatments; (**B**) Shoot dry weight; (**C**) Root dry weight; (**D**) Shoot height; (**E**) Leaf number; (**F**) Branch number; (**G**) Total root length; (**H**) Root surface area; (**I**) Average root diameter. Data in figures are means of five replicates with standard error bars. Asterisks indicate significant difference between pH 5.8 and pH 4.2 treatments in the Student’s *t*-test (*: *p* < 0.05).

Effects of low-pH stress on soybean growth were investigated in soils after 30 days treatment. Results showed that compared to control, soybean root dry weight was decreased by 26% under acid conditions (Figure 1C). Consistently, total root length and root surface area were also significantly decreased by low-pH stress, as reflected by 18% and 17% reduction, respectively (Figure 1G,H). In contrast, no significant differences were observed in shoot dry weight, leaf number, shoot height, branch number and average root diameter between two pH-treatments (Figure 1). The results strongly suggest that soybean roots are more sensitive to low-pH stress than shoots.

### 3.2. Concentration of Mineral Nutrients Affected by Acidity Stress

**Figure 2:**
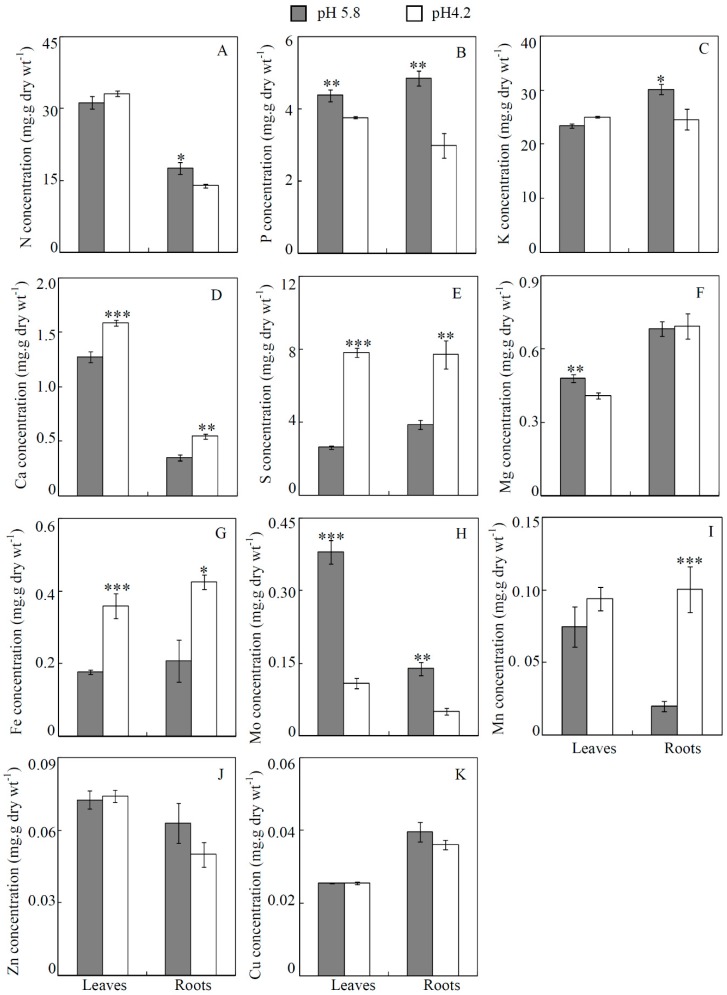
Concentration of mineral nutrients in leaves and roots of soybean plants subjected to two pH-treatments in soil culture. (**A**) N concentration; (**B**) P concentration; (**C**) K concentration t; (**D**) Ca concentration; (**E**) S concentration; (**F**) Mg concentration; (**G**) Fe concentration; (**H**) Mo concentration; (**I**) Mn concentration; (**J**) Zn concentration; (**K**) Cu concentration. Data in figures are means of five replicates with standard error bars. Asterisks indicate significant difference between pH 5.8 and pH 4.2 treatments in the Student’s t-test (*: *p* < 0.05; **: 0.001 < *p* < 0.05; ***: *p* < 0.001).

Concentrations of six mineral macronutrients (i.e., N, P, K, Ca, Mg and S) were separately determined in leaves and roots of soybean grown on soils. It was found that concentrations of the mineral macronutrients were affected by low-pH stress, mainly differing among plant organs and types of mineral nutrients (Figure 2). For N, P, and K, their concentrations in soybean roots were separately decreased by 21%, 38% and 19% by low-pH treatment compared to the control. However, only P concentration was decreased in leaves, while neither N or K concentration in soybean leaves was found to be significantly affected by low-pH stress (Figure 2A–C). Therefore, the results indicate that acidity stress might affect the absorption of K and N, but not their translocation from roots to shoots. 

Concentrations of three other macronutrients (i.e., Ca, Mg and S) were also analyzed in both leaves and roots. Concentrations of Ca and S in both leaves and roots were significantly increased under low-pH conditions (Figure 2D,E). Compared to the control, low-pH treatment increased Ca concentration by 20% in leaves and 35% in roots, respectively (Figure 2D). Similarly, S concentration was increased by 3 folds in leaves and 2 folds in roots, respectively (Figure 2E). However, low-pH led to significant decreases of Mg concertation in leaves, as reflected by 15% reductions, but not in roots (Figure 2F). These results strongly suggest that low pH exhibits different effects of mineral macronutrient acquisition and translocation in soybean. 

Concentrations of five mineral micronutrients, including Fe, Mo, Mn, Zn and Cu, were also determined in soybean leaves and roots at two pH-treatments. It showed that low-pH significantly affected concentrations of Fe, Mo, and Mn, but just slightly influenced the concentrations of Zn and Cu (Figure 2G–K). Under acid conditions, Fe concentration was increased by about 2 folds in both leaves and roots (Figure 2G). Similarly, Mn concentration was increased by about 5 folds in roots by low-pH treatment, whereas, no significant difference was observed in leaves (Figure 2I). In contrast, Mo concentration was significantly reduced under acid conditions, as reflected by 71% decreases in leaves and 64% decreases in roots, respectively (Figure 2H).

### 3.3. Growth and Mineral Nutrient Concentrations in Roots of Soybean Grown in Nutrient Solution

Soybean seedlings were further subjected to hydroponic culture with two pH-treatments (pH 5.8 and pH 4.2) to determine effects of acidity stress on soybean growth and the mineral nutrient concentration in roots. It showed that similar to the results in soil culture, the acidity stress significantly inhibited soybean root growth as indicated by reduced root dry weight and total root length, which were 31% and 19% decreased under acid condition, respectively (Figure S1C,G). However, no significant differences were observed for the shoot dry weight, plant height, leaf number, branch number, root surface area and average root diameter between two pH-treatments (Figure S1). Mineral nutrient concentrations in soybean roots were also measured. Results showed that concentrations of N, P, K and Mo in acid-treated (pH 4.2) soybean roots were 15%, 23%, 28% and 34% less than those of the control (pH 5.8), respectively. In contrast, concentrations of Ca, S and Mn in acid-treated soybean roots were 1.3, 1.5 and 1.9 folds higher than those of control, respectively. These results are similar with that in soil culture. However, no significant differences were observed for concentrations of Fe, Mg, Zn and Cu between two pH-treatments (Figure S2).

### 3.4. Transcriptomic Analysis of Soybean Roots Responding to Acidity Stress

In order to investigate molecular mechanism underlying soybean adaptive strategies, transcriptomic analysis was conducted in soybean roots grown in the nutrient solution at two pH-treatments. For each treatment, three libraries were constructed and produced around 53 million raw reads for each treatment (Table S2). After excluding the low-quality readings, about 44.8 and 44.5 million clean reads were obtained for libraries of control and acid-treated soybean roots, respectively (Table S2). Total number of transcribed genes in control and acid-treated roots were 66,391 and 65,400, respectively (Table S2). Moreover, transcripts resulted in a total of 41,055 and 40,476 known-genes in control and acid-treated roots, respectively (Table S2).

The DEGs between control and acid-treated roots were defined as those with at least 2-fold changes in expression, as well as *p* value 0.05. Using the criteria, a total of 974 genes were considered to be differently expressed in soybean roots between two pH-treatments (Table 1 and Table S3). Among them, 419 genes were up-regulated, while 555 genes were down-regulated by low-pH stress (Table 1 and Table S3). Gene ontology (GO) category analysis showed that 974 DEGs could be divided into 18 biological processes, 14 cellular components, and 11 molecular function terms (Figure S3). The pathway functional enrichment of DEGs showed that most DEGs were predicted to function in the metabolic pathway (Figure S4).

### 3.5. Transcription Factors Involved in Soybean Responses to Acidity Stress

**Figure 3:**
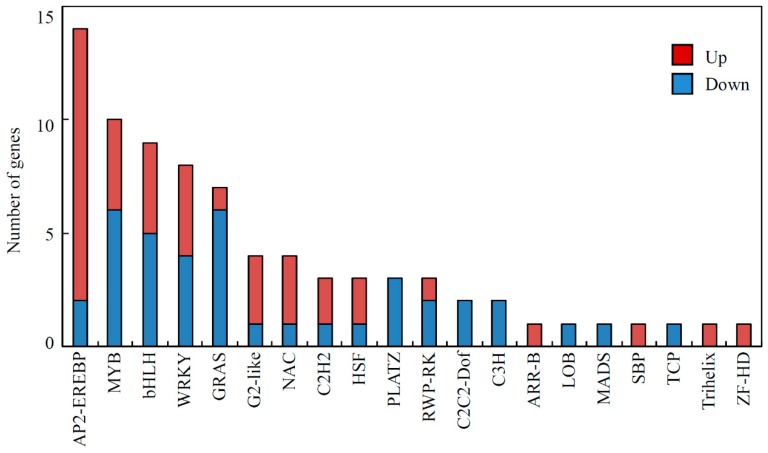
Differentially expressed genes (DEGs) associated with transcription factors in soybean roots.

A total of 79 DEGs were predicted to encode transcription factors (Figure 3, Tables S4 and S5). Among them, genes encoding AP2-EREBP transcription factors were the most abundant, with twelve up-regulated and two down-regulated. Other DEGs encoding transcription factors included ten *MYB*s, nine *bHLH*s, eight *WRK*s, seven *GRAS*s, four *G2-likes* and *NAC*s, three *C2H2*s, *HSF*s, *PLATZ*s, and *RWP-RK*s, two *C2C2-Dofs* and *C3H*s, as well as one *MADS*, *LOB*, *TCP*, *ARR-B*, *SBP*, *Trihelix*, *and ZF-HD* (Figure 3 and Table S4). 

Among them, two up-regulated *C2H2* genes, Glyma.12G081700 (*GmSTOP2-1*) and Glyma.11G192400 (*GmSTOP2-2*), are *AtSTOP2* homologues. Under low-pH conditions, expression of *GmSTOP2-1* and *GmSTOP2-2* was increased by about 8 and 10 folds, respectively (Table S4). In contrast, one of *MYB* genes, Glyma.02G177800, was predicted to encode a *PHR1-LIKE* (*PHL*) transcription factor, whose expression level was decreased by 83% under acid conditions (Table S4).

### 3.6. Identification of DEGs Involved in the pH Stat Pathway

A set of DEGs was predicted to be involved in the pH homeostasis process in plant cells, namely pH stat pathway [58]. The genes included one glutamate dehydrogenases (i.e., *GmGDH1*), two malic enzymes (i.e., *GmME1-1/2*), three pyruvate decarboxylases (i.e., *GmPCD1* and *GmPCD2-1/2*) and four alcohol dehydrogenases (i.e., *GmADH1-1/2/3* and *GmADH4*) (Table 2). Most of these genes were up-regulated by acidity stress, except for *GmME1-1* and *GmADH4*, whose expression was suppressed by 2.8 and 2.5 times under acid conditions. Among the up-regulated DEGs, transcript level of *GmGDH1*, which was involved in the GABA shunt pathway, was increased by about 3.5 folds (Table 2). Moreover, expression level of *GmME1-2* and *GmADH1-2* was separately increased by about 5.3 and 6.5 folds (Table 2). These results indicate that pH stat pathway is significantly influenced in soybean roots by acidity stress.

### 3.7. DEGs Participated in Mineral Nutrients Acquisition and Translocation

A total of thirty-eight DEGs were found to be related to mineral nutrients acquisition and translocation, of which twenty-nine genes were down-regulated and nine genes were up-regulated (Table 3 and Table S5). These DEGs encoded two ammonium transporters (*GmAMT1/2*), eight nitrate transporters (*GmNPF7.3-1/2*, *GmNRT2*, *GmNRT2.4-1/2*, *GmNRT2.5*, *GmNPF3.1/4.6*), five phosphate transporters (*GmPT1/3/4/7/13*), six putative potassium transporters (*GmHKT1*, *GmKUP3/6*, *GmTPK5*, *GmSKOR*, *GmKT2*), eight sulfate transporters (*GmSULRT2.1-1/2/3*, *GmSULRT3.1-1/2/3*, *GmSULRT3.5-1/2*), four zinc transporters (*GmZIP1/2*, *GmZIP11-1/2*) and five heavy mental transporters (*GmMTP1-1/2*, *GmNramp5*, *GmIRT1*, *GmVIT2.1*). Among them, all *GmAMTs*, *GmPTs*, *GmKUPs, and GmZIPs*, together with *GmIRT1*, *GmNramp5*, *GmVIT2.1*, *GmSKOR*, *GmTPK5* and *GmHKT1*, were significantly down-regulated in soybean roots by low-pH treatments (Table 3). 

However, members of *GmNRT/NPF*, *GmSULTR* and *GmMTP* family were found either down-regulated or up-regulated by acidity stress (Table 3). It showed that for eight *GmNRT* members, transcripts of six members were down-regulated, while two were up-regulated (Table 3). Among the six down-regulated *GmNRT* members, four members (i.e., *GmNRT2.5*, *GmNRT2.4-1*, *GmNRT2.4-2 and GmNRT2*) belonged to the high affinity nitrate transporter, and two (i.e., *GmNPF7.3-1* and *GmNPF7.3-2*) belonged to the dual-affinity nitrate transporter. Similarly, for *SULTR* family, all of three *SULTR2* members, including *GmSULTR2.1-1/2/3*, were down-regulated. In contrast, all of five *SULTR3* members, including three *GmSULTR3.1* members (i.e., *GmSULTR3.1-1/2/3*) and two *GmSULTR3.5* members (i.e., *GmSULTR3.5-1/2*) were up-regulated under acid conditions (Table 3). Identification of DEGs associated with mineral nutrients acquisition and translocation strongly indicates that changes of mineral nutrient concentrations in soybean leaves and roots might be regulated by these DEGs under low-pH conditions.

### 3.8. Transcripts Analysis of DEGs Involved in Nutrients Transportation Using qRT-PCR

**Figure 4:**
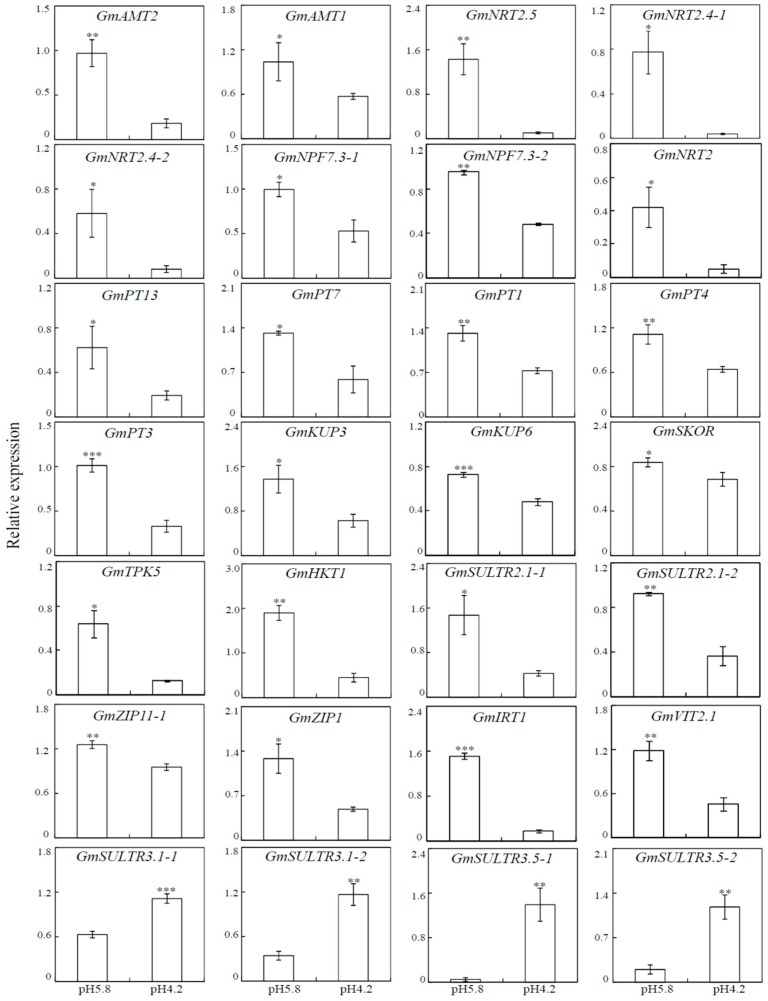
qRT-PCR analysis of DEGs transcription in soybean roots. Data in the figure are means of three replicates with standard error bars. Asterisks indicate significant difference between pH 5.8 and pH 4.2 treatments in the Student’s t-test (*: *p* < 0.05; **:0.001 < *p* < 0.05; ***: *p* < 0.001).

To confirm transcriptome results, qRT-PCR analysis was further conducted to investigate the expression of sets of DEGs involved in mineral nutrient acquisition and translocation, including twenty-four down-regulated and four up-regulated genes. Results showed a significant correlation presented between transcriptome and qRT-PCR analyses (r = 0.713, *p* < 0.01) (Figure 4 and Figure S5). Among down-regulated genes, it was observed that expression of *GmNRT2.4-1* and *GmNRT2* was decreased by 19-and 8-folds, respectively (Figure 4). For the four up-regulated genes (i.e., *GmSULTR3.1-1/2* and *GmSULTR3.5-1/2*), fold changes of their expression ranged from 1.7 to 7.4 (Figure 4).

## 4. Discussion

Low pH is a major constraint on crop productivity in the world’s acid soil regions [2]. It has been documented that low soil pH not only limits nutrient availability, but also increases metal toxicity underground [21,59]. This poor growth condition leads to yield losses of more than 50% in grain crops [10]. So far, most studies have been focusing on plant growth limitations caused by P deficiency, or metal toxicities (i.e., Al and Mn) in acid soils [2,60]. However, the adverse effects of acidity stress alone, which is equally considered as a limiting factor for plant growth in acid soils, are often less emphasized [33,61]. In the study, effects of low-pH on soybean growth and its mineral nutrient homeostasis were investigated. Subsequently, transcriptomic analyses in soybean roots were conducted to shed light on molecular mechanisms underlying adaptation of soybean to acidity stress, especially mineral nutrients acquisition and translocation.

It showed that after the acid treatment, soybean root growth was significantly inhibited, as indicated by reduced root dry weight and total root length in both soil and hydroponic cultures (Figure 1 and Figure S1). This is consistent with previous reports in Arabidopsis, field beans (*Vicia faba*), corn (*Zea mays*) and lotus, in which significant decreases of root growth were found to be the main symptoms of proton toxicity [12,13,14,15,62]. Since mineral nutrients are mainly taken up by roots, it is generally assumed that mineral nutrient acquisition and translocation must be severely inhibited in plants under low-pH conditions. However, the present results showed that most essential mineral nutrients in soybean leaves and roots were differentially accumulated and tightly dependent on soybean organs and mineral nutrient types (Figure 2 and Figure S2). For examples, the N, P, K, and Mo concentrations in soybean roots were decreased, while concentrations of Ca, S, Fe and Mn were increased under acid conditions in soil culture (Figure 2). In the hydroponic culture, similar results were also observed in soybean roots, except for Fe concentration, which was slightly increased but not significant (Figure S2). Other study of Erica also showed that acidity stress significantly affected the Mg and Mn concentrations in leaves, S, B and Fe concentrations in stems, and Ca, P, Fe, Cu and Zn concentrations in roots [25]. Thus, these results strongly suggested that the capability of mineral nutrients acquisition and translocation in plants could be influenced by proton toxicity.

To understand the molecular mechanisms underlying genes regulation in soybean responses to acidity stress, the transcriptome assay of soybean roots was conducted. It showed that long-term acidity stress mainly affects the expression of genes function in metabolic pathways (Figure S4). Moreover, a proportion of low-pH up-regulated genes are categorized as pathogen response genes, including genes involved in glycollins biosynthesis, such as *C4H* (Glyma.20G114200), *CHR* (Glyma.02G307300), *G4DT* (Glyma.10G295300) and genes involved in SAR, such as *DIR1* (Glyma.11G120400), *WRKY40* (Glyma.14G103100), *RLP32* (Glyma.16G169500), etc. (Table S3). This is consistent with the recent report that these genes were up-regulated in soybean variety Harosoy 63 after a 9-day pH3.0 treatment [35]. Therefore, it suggests that overlays between acidity-stress responses and pathogen responses are conserved in soybean.

Moreover, a total of thirty-eight DEGs associated with nutrient acquisition and transportation were identified, indicating that acid-responsive mineral nutrient homeostasis in soybean might be affected by the regulation of DEGs encoding mineral nutrient transporters (Table 3). For example, ten DEGs were identified to be involved in N transportation, including two AMTs, four NPFs and four NRTs (Table 3). It has been well documented that AMT1 and AMT2 proteins play critical roles in ammonium uptake and root-to-shoot translocation, respectively [30,63,64,65,66,67]. Moreover, NPF and NRT2 are two major transporter families for NO_3_^−^ absorption and transportation in plant species [68,69,70,71]. Our results showed that except for *GmNPF3.1* and *GmNPF4.6*, all the other eight DEGs encoding NO_3_^−^ transporters were significantly down-regulated, which exhibited a positive correlation with the reduced N concentration in low-pH-treated soybean roots (Figure 2 and Table 3). Similar results were also found for P and K concentrations and expression of their corresponding transporter genes. It showed that P and K concentrations were significantly decreased in acid-treated soybean roots (Figure 2). Meanwhile, all five Pi transporter genes (i.e., *GmPT1/3/4/7/13*) and five K transporter genes (i.e., *GmHKT1*, *GmKUP3/6*, *GmTPK5*, *GmSKOR*) were also down-regulated by acid-treatment (Table 3). Although functions of most Pi transporter and K transporter genes remain largely unknown in soybean, five *GmPT*s were suggested to encode high-affinity Pi transporters through complementary experiments in yeasts [72]. Meanwhile, the homologues of *HKT*, *KUP*, *TPK,* and *SKOR* have been demonstrated to function in K^+^ absorption or translocation in other plant species [73,74,75,76]. Therefore, it is suggested that down-regulated expression of DEGs involved in N, P, and K transportation might play important roles in mediating N, P, and K homeostasis in soybean under acid conditions.

In addition, eight *GmSULTR* genes were identified in the transcriptome assay, which were classified into *GmSULTR2* and *GmSULTR3* sub-groups [77] (Table 3). Transcripts of all three *GmSULTR2*s were down-regulated by acid treatments (Figure 4 and Table 3). Phylogenetic analysis showed that three GmSULTR2 members shared high similarity with SHST1, a putative sulfate transporter in Stylosanthes hamata that also could transport Mo [78]. Therefore, it was suggested that suppressed expression of *GmSULTR2* genes might be responsible for the decreased Mo concentration in the acid-treated soybean roots (Figure 2). In contrast, five *GmSULTR3* genes were all up-regulated by acid-treatments (Table 3). The phylogenetic analysis showed high similarity between GmSULTR3.1/5 and AtSULTR3;1/5 in Arabidopsis (Figure S6). It has been suggested that *AtSULTR3;1* was responsible for sulfate transportation into chloroplasts [79], while *AtSULTR3;5* was involved in root-to-shoot sulfate transportation and sulfate translocation within developing seeds [80,81]. Taken together with the increased sulfate accumulation in soybean plants under acid conditions (Figure 2 and Figure S2), it was indicated that up-regulated GmSULTR3 genes might play roles in the translocation or compartmentalization of excessive sulfate in roots.

Several metal transporter genes, including *GmZIP1/2*, *GmZIP11-1/2*, *GmNramp5* and *GmMTP7-1/2*, were found to exhibit differential expression patterns under acid conditions. The homologues of ZIP and Nramp5 in other plant species have been suggested to mediate Mn uptake and/or translocation [82,83,84,85,86]. Meanwhile, phylogenetic analysis showed that GmMTP7-1/2 shared high similarity with Mn-CDF MTP proteins in rice and Arabidopsis (Figure S7), which function in Mn acquisition and translocation [87,88]. Combining the higher Mn concentration in acid-treated soybean roots (Figure 2 and Figure S2), it therefore suggests that suppressed expression of DEGs involving in Mn transportation might play roles in reducing Mn content in soybean roots under acid conditions.

Besides the DEGs involving in nutrient transportation, ten DEGs were identified to be associated with plant pH stat pathways (Table 2). One of these pH stat pathways is the biochemical pH stat pathway, which plays important role in the H^+^ consumption, and consists of alcohol dehydrogenase, malic enzyme and pyruvate decarboxylase [58,89,90]. The other pH stat pathway is the GABA shunt pathway, which consists of GDH, GAD, and GABA-T and also contributes greatly to the H^+^ homeostasis in plant cells [58,91,92]. Expression of genes involving in these two pH-stat pathways was significantly suppressed in Arabidopsis stop1 mutants, strongly suggesting that they play important roles in Arabidopsis low-pH-tolerance [35]. In the present study, GmGDH1 in the GABA shunt pathway, and several other genes (i.e., *GmME1-1*, *GmPCD1*, *GmPCD2-1/2*, *GmADH1-1/2/3*) in the other biochemical pH stat pathway were significantly up-regulated under acid conditions (Table 2). Taken together, our results suggested that genes involving in pH-stat pathways could contribute greatly to the low-pH tolerance in soybean. The functions of DEGs associated with pH-stat pathways merit further characterization.

Finally, the identification of 79 transcription factors responding to low-pH treatment further shed light on the complex regulatory networks in soybean root responses to acidity stress (Table S4). It was recently reported that a NAC family transcription factor *GmNAC42-1* (Glyma.02G284300), together with its down-stream genes *IFS2* (Glyma.13G173500) and *G4DT* (Glyma.10G295300) was significantly up-regulated by 9-day low-pH treatment, indicating a regulatory link between the acidity and pathogen responses in soybean [35]. However, our transcripteomic data showed that the expression of *GmNAC42-1* and *IFS2* was no affected, while *G4DT* was up-regulated by the long-term acidity stress (30day) (Tables S3 and S4). Therefore, it indicates that there are might be other regulatory pathways involved in the pathogen responses under the long-term acid condition. Moreover, a *PHR1-LIKE1* (*GmPHL1*) was down-regulated under acid conditions (Table S4). Although the function of *GmPHL1* remains largely unknown in soybean, homologue of *PHL1* in Arabidopsis is suggested to be closely related to *PHR1* and act redundantly to regulate plant responses to Pi starvation [93]. It thus suggests that *GmPHL1* might be involved in Pi homeostasis in response to acidity stress in soybean roots. Moreover, expression levels of two C2H2 transcription factors, *GmSTOP2*s, were up-regulated by acidity stress in soybean (Table S4). Since it is well known that *AtSTOP2* in Arabidopsis partially confers low pH-tolerance by recovering expression of genes regulated by *AtSTOP1* [94], our results indicate that *GmSTOP2* genes might play important roles in soybean acid tolerance.

Overall, the present study showed that long-term acidity stress significantly inhibited soybean root growth and remarkably affected soybean mineral nutrients accumulation. Consistently, our transcriptome and qRT-PCR analyses showed that a set of DEGs might play important roles in mediating mineral nutrient homeostasis in soybean responses to acidity stress. Moreover, identification of DEGs associated with pH stat pathway strongly suggested that maintaining pH stat is one adaptive strategy of soybean responses to acidity stress. Taken together, the results herein provided new insights into physiological and molecular mechanisms underlying soybean adaptation to acidity stress.

## Figures and Tables

**Table 1:** Gene number identified in soybean roots with two pH-treatments.

	Total Expressed Genes	Up-Regulated Genes	Down-Regulated Genes
pH 5.8	42,621		
pH 4.2	41,857		
DEGs *	974	419	555

* DEGs: Differentially expressed genes between control (pH 5.8) and acid-treated (pH 4.2) soybean roots.

**Table 2:** DEGs related to pH stat pathway in soybean roots.

Number	Gene ID	Expression Level at pH 5.8	Expression Level at pH 4.2	Log_2_ Fold Change (pH 4.2/pH 5.8)	*p* Value	Description	Gene Name
1	Glyma.16G041200	243.18	823.46	1.8	8.49 × 10^−12^	glutamate dehydrogenase 1	*GmGDH1*
2	Glyma.09G262900	122.93	43.47	−1.5	2.20×10^−^^5^	NAD-dependent malic enzyme	*GmME1-1*
3	Glyma.04G086300	37.51	198.71	2.4	2.91×10^−^^6^	NAD-dependent malic enzyme	*GmME1-2*
4	Glyma.13G231700	972.9	3302.8	1.8	1.74×10^−^^6^	pyruvate decarboxylase 1	*GmPCD1*
5	Glyma.18G204200	1282.4	3518.3	1.5	2.88×10^−^^5^	pyruvate decarboxylase 2	*GmPCD2-1*
6	Glyma.07G153100	934.5	3283.6	1.8	1.57×10^−^^5^	pyruvate decarboxylase 2	*GmPCD2-2*
7	Glyma.04G240800	1201.0	4375.6	1.9	5.47×10^−^^8^	alcohol dehydrogenase 1	*GmADH1-1*
8	Glyma.14G121200	63.3	399.0	2.7	5.31×10^−^^16^	alcohol dehydrogenase 1-like	*GmADH1-2*
9	Glyma.06G122600	3010.3	10795.3	1.8	2.14×10^−^^7^	alcohol dehydrogenase 1	*GmADH1-3*
10	Glyma.18G200300	467.7	187.2	−1.3	3.59×10^−^^5^	alcohol dehydrogenase-like 4	*GmADH4*

**Table 3:** DEGs related to nutrients transportation in soybean roots.

Number	Gene ID	Expression Level at pH5.8	Expression Level at pH4.2	Log_2_ Fold Change (pH 4.2/pH 5.8)	*p* Value	Description	Gene Name
1	Glyma.10G132300	306.82	127.02	−1.27	2.8 × 10^−^^4^	ammonium transporter 1	*GmAMT1*
2	Glyma.01G123400	81.10	6.82	−3.57	6.37 × 10^−^^11^	ammonium transporter 2-like	*GmAMT2*
3	Glyma.05G070600	194.19	62.38	−1.64	1.25 × 10^−^^4^	protein NRT1/PTR FAMILY 7.3-like	*GmNPF7.3-1*
4	Glyma.17G153300	611.91	209.01	−1.55	9.05 × 10^−^^4^	protein NRT1/PTR FAMILY 7.3-like	*GmNPF7.3-2*
5	Glyma.13G323800	5449.11	476.64	−3.52	3.00 × 10^−^^23^	NRT2 protein	*GmNRT2*
6	Glyma.12G176900	2041.80	13.51	−7.24	6.20 × 10^−^^66^	high affinity nitrate transporter 2.4-like	*GmNRT2.4-1*
7	Glyma.11G195200	8226.27	426.09	−4.27	4.28 × 10^−^^31^	high affinity nitrate transporter 2.4	*GmNRT2.4-2*
8	Glyma.18G141900	1306.71	33.46	−5.29	1.53 × 10^−^^33^	high affinity nitrate transporter 2.5-like	*GmNRT2.5*
9	Glyma.03G122500	352.71	882.01	1.32	8.93 × 10^−^^4^	protein NRT1/PTR FAMILY 3.1	*GmNPF3.1*
10	Glyma.18G126500	23.47	77.52	1.72	4.03 × 10^−^^5^	protein NRT1/PTR FAMILY 4.6	*GmNPF4.6*
11	Glyma.02G005800	665.66	6.09	−6.77	1.42 × 10^−^^5^	inorganic phosphate transporter 1-7-like	*GmPT1*
12	Glyma.07G222700	188.38	63.69	−1.56	2.98 × 10^−^^5^	inorganic phosphate transporter 1-9-like	*GmPT3*
13	Glyma.10G006700	1478.73	379.51	−1.96	4.63 × 10^−^^6^	inorganic phosphate transporter 1-3	*GmPT4*
14	Glyma.10G186500	919.56	381.20	−1.27	1.82 × 10^−^^4^	inorganic phosphate transporter 1-7-like	*GmPT7*
15	Glyma.20G204000	294.20	71.42	−2.04	2.17 × 10^−^^5^	inorganic phosphate transporter 1-7-like	*GmPT13*
16	Glyma.12G133400	96.70	22.67	−2.09	1.87 × 10^−^^7^	sodium transporter HKT1-like	*GmHKT1*
17	Glyma.16G046200	669.36	304.90	−1.13	3.12 × 10^−^^5^	potassium transporter 4-like	*GmKUP3*
18	Glyma.02G033600	566.51	275.46	−1.04	2.81 × 10^−^^4^	Potassium transporter 6	*GmKUP6*
19	Glyma.03G223900	43.03	17.06	−1.33	1.01 × 10^−^^3^	two-pore potassium channel 5-like	*GmTPK5*
20	Glyma.02G243400	692.88	298.10	−1.21	3.79 × 10^−^^5^	potassium channel SKOR-like	*GmSKOR*
21	Glyma.06G143800	100.89	249.30	1.31	4.16 × 10^−^^6^	potassium transporter 2-like	*GmKT2*
22	Glyma.11G238400	70.15	18.35	−1.93	2.75 × 10^−^^6^	sulfate transporter 2.1-like	*GmSULTR2.1-1*
23	Glyma.08G138600	78.51	29.44	−1.42	1.81 × 10^−^^4^	sulfate transporter 2.1-like	*GmSULTR2.1-2*
24	Glyma.18G019000	123.53	26.66	−2.21	6.41 × 10^−^^8^	sulfate transporter 2.1-like	*GmSULTR2.1-3*
25	Glyma.19G159000	126.03	411.14	1.71	4.51 × 10^−^^6^	sulfate transporter 3.1-like	*GmSULTR3.1-1*
26	Glyma.02G145100	83.76	214.91	1.36	1.38 × 10^−^^6^	sulfate transporter 3.1-like	*GmSULTR3.1-2*
27	Glyma.03G156700	378.82	1484.45	1.97	2.27 × 10^−^^6^	sulfate transporter 3.1-like	*GmSULTR3.1-3*
28	Glyma.09G188700	4.38	64.18	3.87	4.31 × 10^−1^^3^	sulfate transporter 3.5	*GmSULTR3.5-1*
29	Glyma.07G088200	89.10	448.25	2.33	7.3 × 10^−^^4^	sulfate transporter 3.5	*GmSULTR3.5-2*
30	Glyma.13G004400	1633.97	480.53	−1.77	7.75 × 10^−^^11^	zinc transporter 1-like isoform X2	*GmZIP1*
31	Glyma.13G338300	495.89	159.55	−1.64	2.28 × 10^−^^7^	zinc transporter 1-like	*GmZIP2*
32	Glyma.08G328000	77.30	20.91	−1.89	2.66 × 10^−^^5^	zinc transporter 11	*GmZIP11-1*
33	Glyma.15G036200	208.80	85.91	−1.28	3.16 × 10^−^^5^	Zinc transporter 1	*GmZIP11-2*
34	Glyma.09G122600	469.38	132.66	−1.82	1.22 × 10^−^^4^	metal tolerance protein 10-like	*GmMTP7-1*
35	Glyma.08G164800	71.85	220.35	1.62	7.03 × 10^−^^7^	metal tolerance protein 10-like	*GmMTP7-2*
36	Glyma.06G115800	1674.89	353.75	−2.24	3.48 × 10^−^^6^	metal transporter Nramp5-like	*GmNramp5*
37	Glyma.07G223200	10.03	1.80	−2.48	2.79 × 10^−^^4^	zinc transporter 10-like precursor	*GmIRT1*
38	Glyma.05G121300	68.95	21.61	−1.67	1.65 × 10^−^^4^	vacuolar iron transporter homolog 4-like	*GmVIT2.1*

## Supplementary Materials

The following are available online at https://www.mdpi.com/2073-4425/10/5/402/s1, Figure S1. Effects of acidity stress on soybean growth in hydroponic culture. (A) Phenotype of soybean plants at two pH levels; (B) Shoot dry weight; (C) Root dry weight; (D) Shoot height; (E) Leaf number; (F) Branch number; (G)Total root length; (H) Root surface area; (I) Average root diameter. Data in figures are means of three replicates with standard error bars. Asterisks indicate significant difference between pH5.8 and pH4.2 treatments in the Student’s t-test (*: *p* < 0.05). Figure S2. Concentration of mineral nutrients in leaves and roots of soybean subjected to two pH treatments in hydroponic culture. Data in figures are means of three replicates with standard error bars. Asterisks indicate significant difference between pH 5.8 and pH 4.2 treatments in the Student’s t-test (*: *p* < 0.05; **: 0.001 < *p* < 0.05). Figure S3. The GO analysis of DEGs in soybean roots. Figure S4. Pathway functional enrichment of DEGs in soybean roots. Figure S5. Correlation of gene expression levels between transcriptome data and qRT-PCR analysis. Figure S6. Phylogeny analysis of sulfate transporter proteins. Figure S7. Phylogeny analysis of metal tolerance proteins. Table S1. Primers used for qRT-PCR. Table S2. Summary of gene expression. Table S3. General information about DEGs between control (pH 5.8) and acid-treated (pH 4.2) soybean roots. Table S4. Transcription factors respond to acidity stress in soybean roots. Table S5. Abbreviations of DEGs.
