# Supplementary figures and images for: Complex Gene Regulation Underlying Mineral Nutrient Homeostasis in Soybean Root Response to Acidity Stress
Source: Genes (Basel). 2019 May 27;10(5):402. doi: 10.3390/genes10050402 (PMC6563148; doi:10.3390/genes10050402)

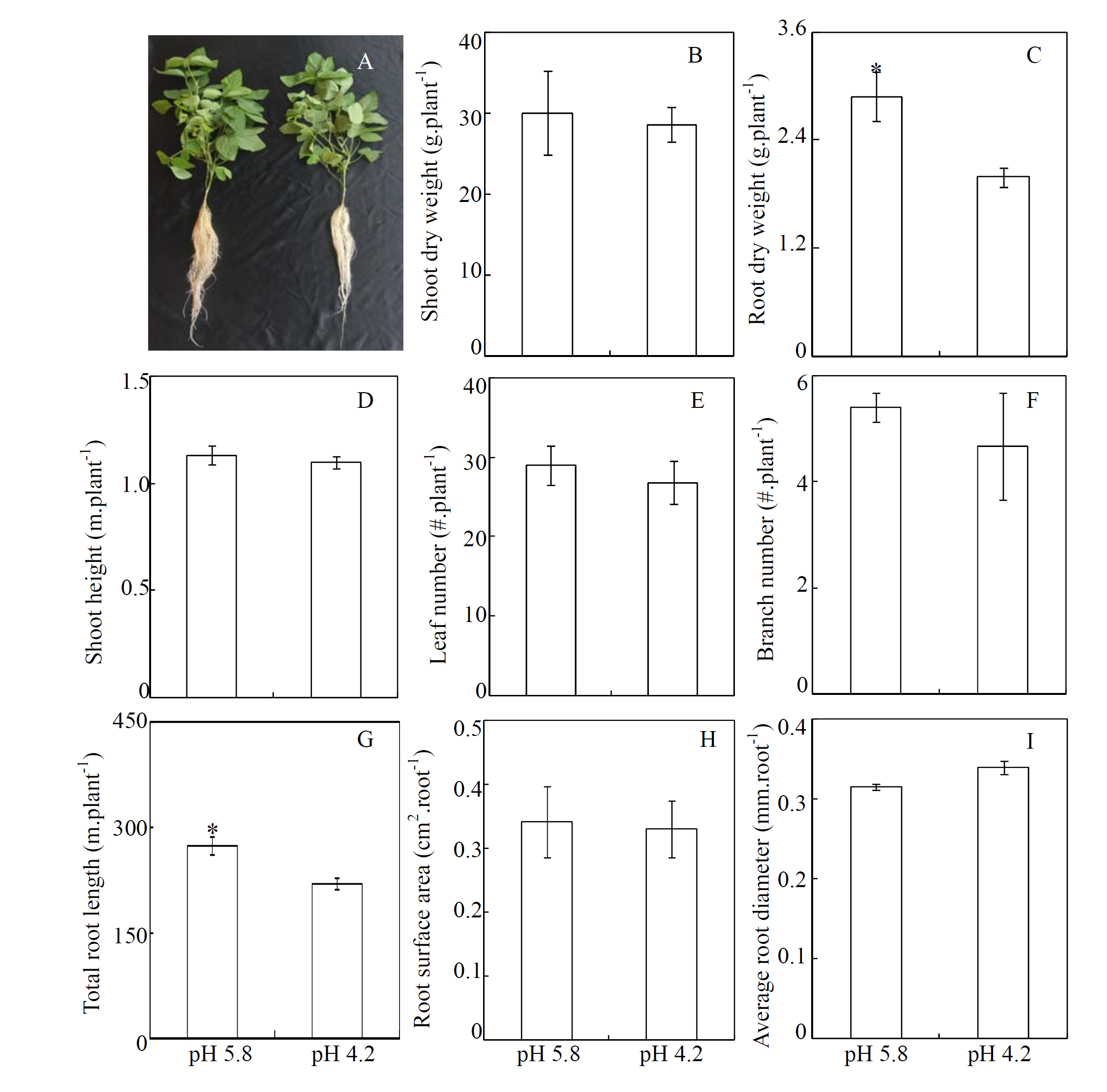

Supplement: Supplementary file 1 [file genes-10-00402-s001.zip › figure S1.tif]

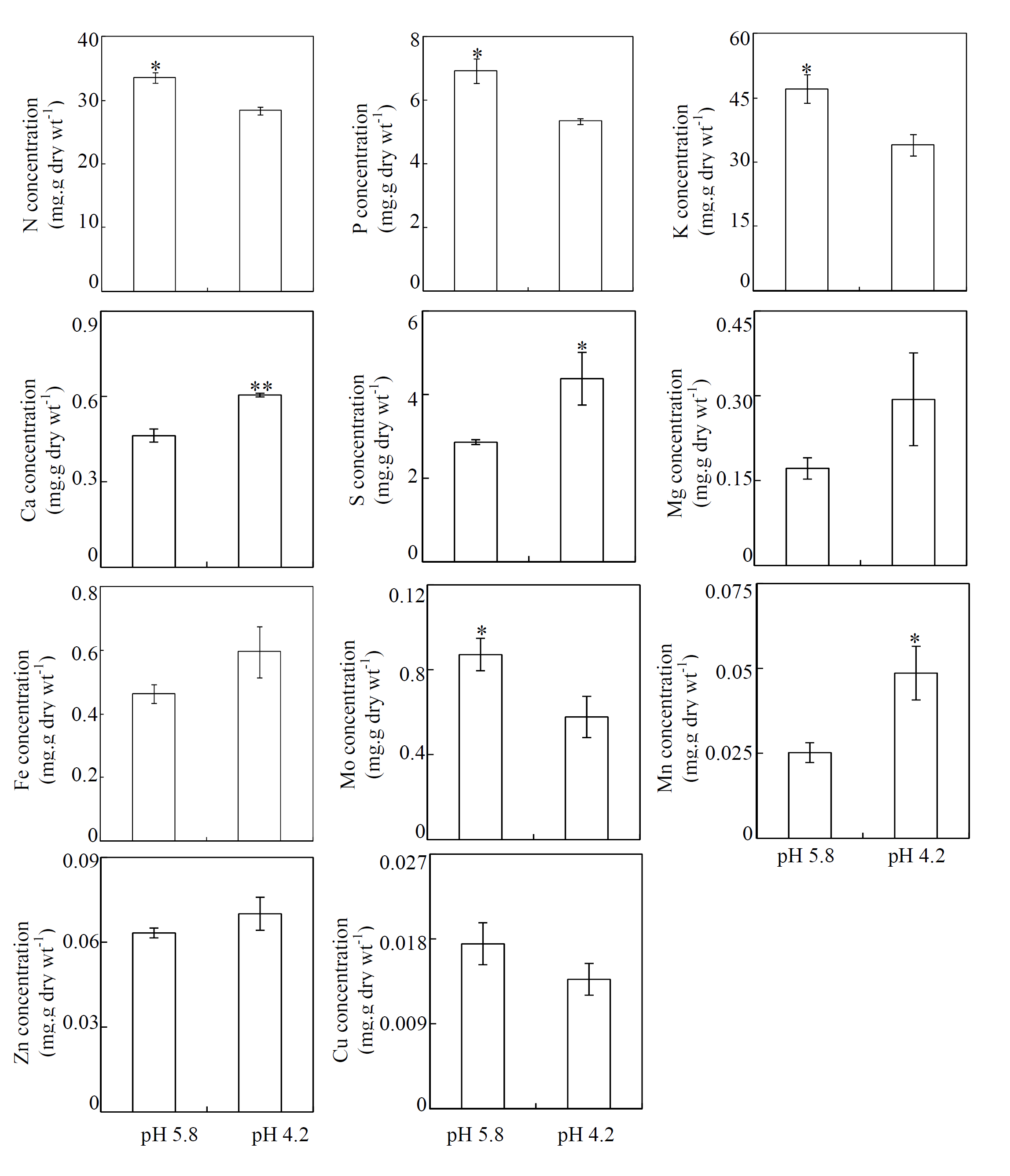

Supplement: Supplementary file 1 [file genes-10-00402-s001.zip › figure S2.tif]

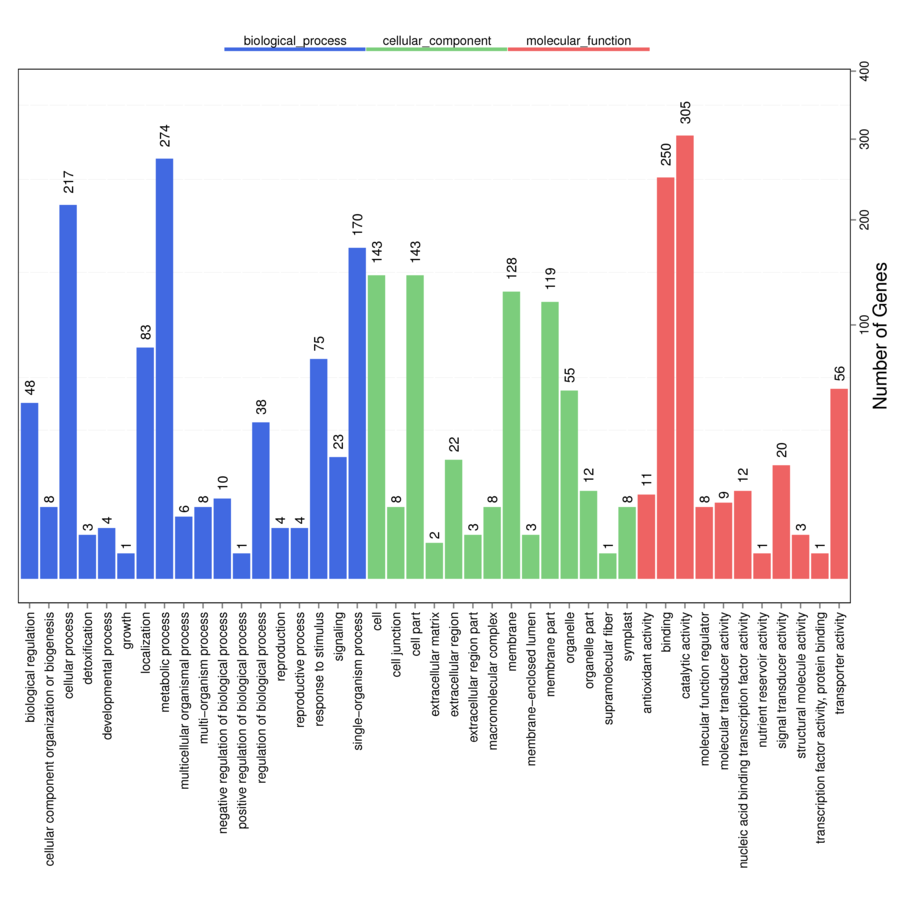

Supplement: Supplementary file 1 [file genes-10-00402-s001.zip › figure S3.tif]

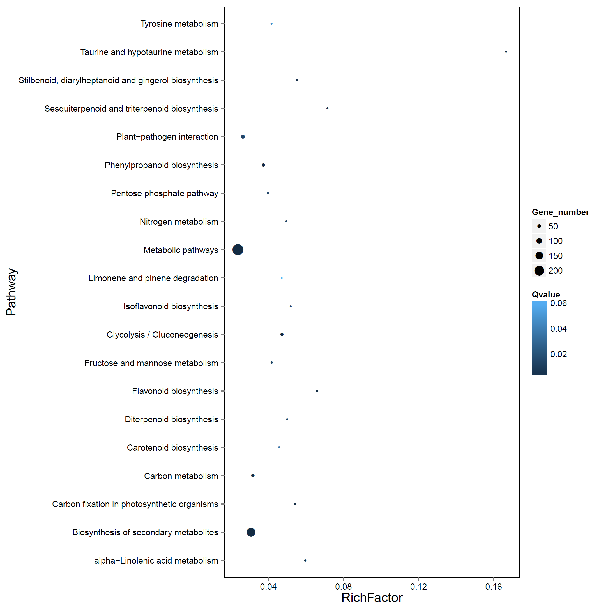

Supplement: Supplementary file 1 [file genes-10-00402-s001.zip › figure S4.tif]

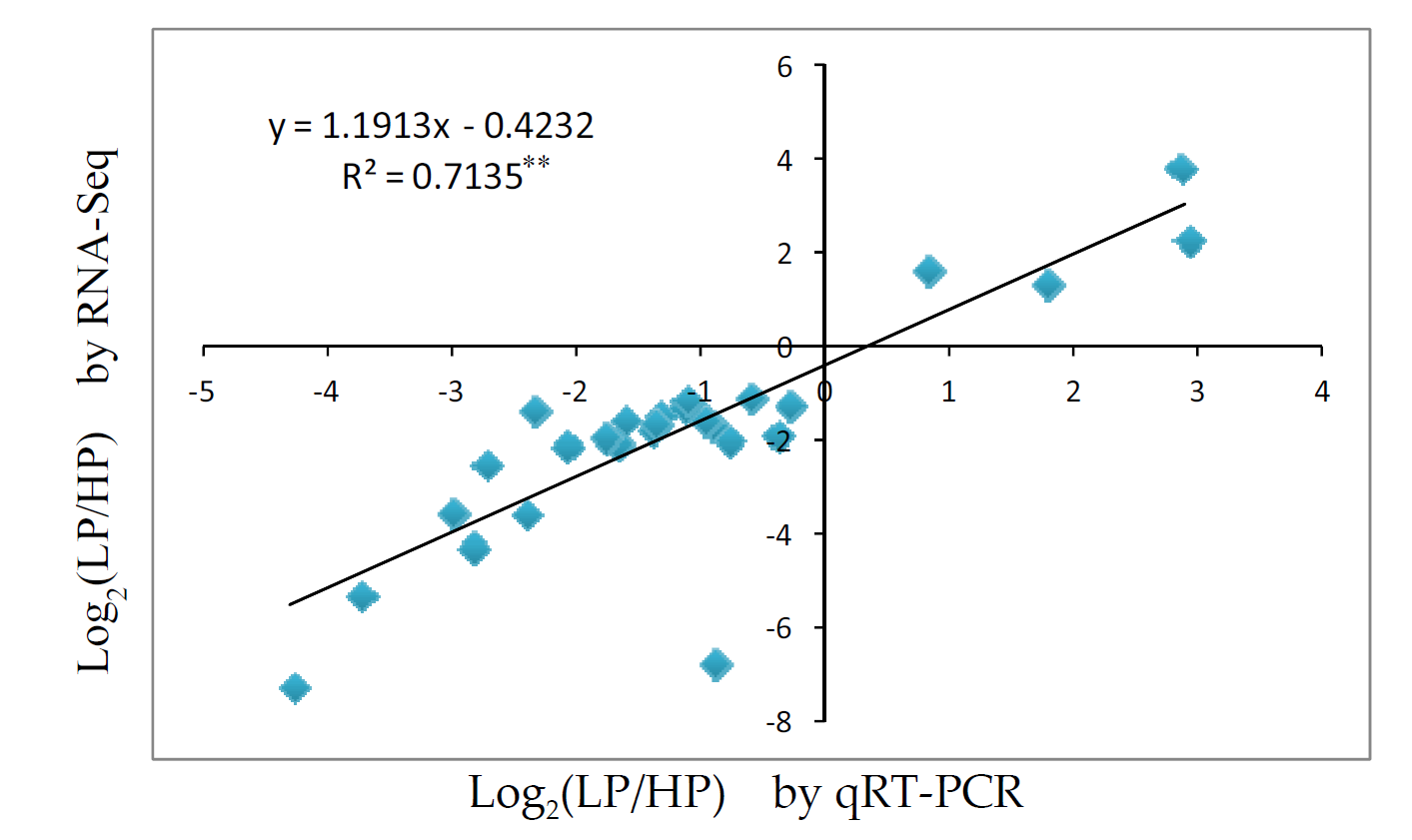

Supplement: Supplementary file 1 [file genes-10-00402-s001.zip › figure S5.tif]

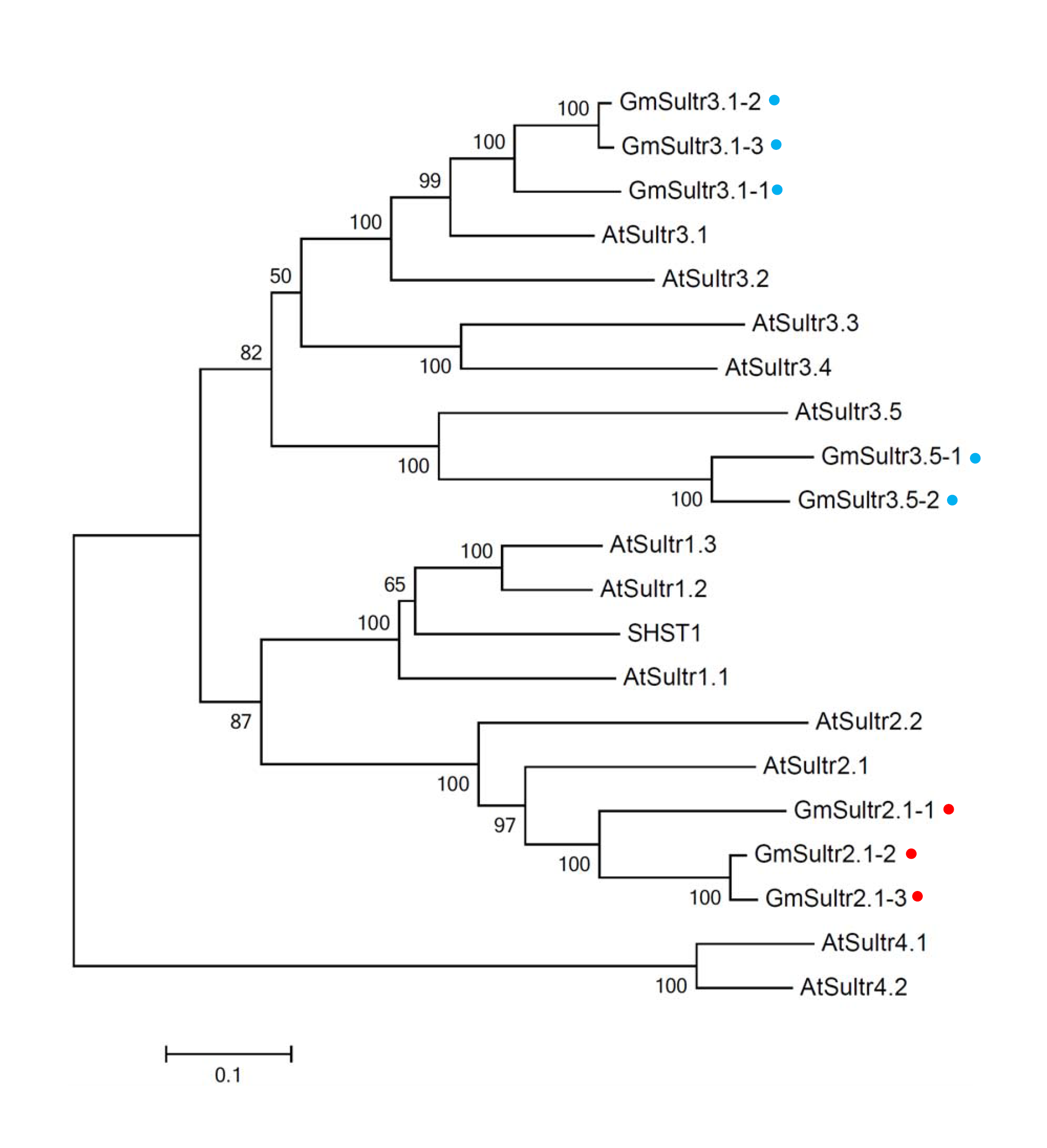

Supplement: Supplementary file 1 [file genes-10-00402-s001.zip › figure S6.tif]

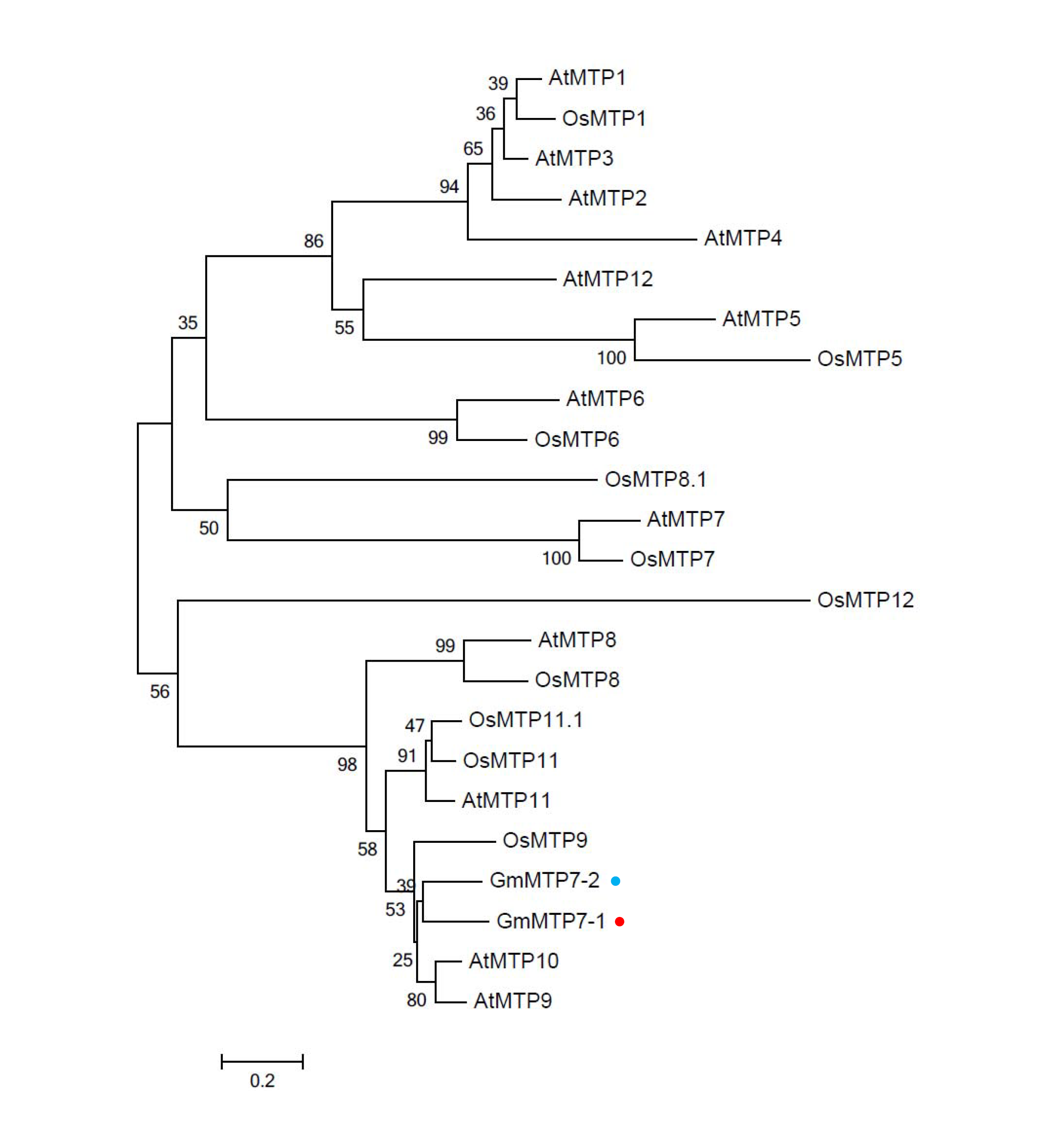

Supplement: Supplementary file 1 [file genes-10-00402-s001.zip › figure S7.tif]
